# Supplementary material for: Mobile Laboratory Reveals the Circulation of Dengue Virus Serotype I of Asian Origin in Medina Gounass (Guediawaye), Senegal
Source: Diagnostics (Basel). 2020 Jun 16;10(6):408. doi: 10.3390/diagnostics10060408 (PMC7345902; doi:10.3390/diagnostics10060408)
Supplement: Supplementary file 1 [file diagnostics-10-00408-s001.zip › Supplementary/Figure S1.pdf]

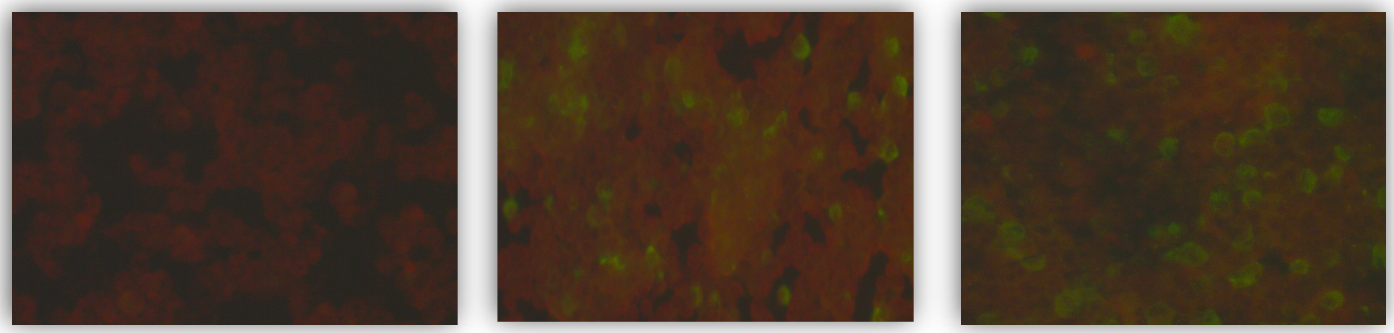

A B C

Figure S1: Assessment of Dengue virus infection using immunofluorescence Indirect Assay (IFA) on C6/36 cells infected with 200 µl of crushed mouse brain previously inoculated with Positive Sera. Immune Ascite fluid were used as Primary antibody (A: Negative control, B : Medina Gounass 1, C : Medina Gounass 2)
